# Antimicrobial Activities of Leaf Extracts of Guava (*Psidium guajava* L.) on Two Gram-Negative and Gram-Positive Bacteria

**DOI:** 10.1155/2013/746165

**Published:** 2013-10-20

**Authors:** Bipul Biswas, Kimberly Rogers, Fredrick McLaughlin, Dwayne Daniels, Anand Yadav

**Affiliations:** ^1^MS Biotechnology Program, College of Agriculture, Family Sciences and Technology, Fort Valley State University, Fort Valley, GA 31030, USA; ^2^Department of Biology, Fort Valley State University, Fort Valley, GA 31030, USA; ^3^Department of Chemistry, Fort Valley State University, Fort Valley, GA 31030, USA

## Abstract

*Aim.* To determine the antimicrobial potential of guava (*Psidium guajava*) leaf extracts against two gram-negative bacteria (*Escherichia coli* and *Salmonella enteritidis*) and two gram-positive bacteria (*Staphylococcus aureus* and *Bacillus cereus*) which are some of foodborne and spoilage bacteria. The guava leaves were extracted in four different solvents of increasing polarities (hexane, methanol, ethanol, and water). The efficacy of these extracts was tested against those bacteria through a well-diffusion method employing 50 **μ**L leaf-extract solution per well. According to the findings of the antibacterial assay, the methanol and ethanol extracts of the guava leaves showed inhibitory activity against gram-positive bacteria, whereas the gram-negative bacteria were resistant to all the solvent extracts. The methanol extract had an antibacterial activity with mean zones of inhibition of 8.27 and 12.3 mm, and the ethanol extract had a mean zone of inhibition of 6.11 and 11.0 mm against *B. cereus* and *S. aureus*, respectively. On the basis of the present finding, guava leaf-extract might be a good candidate in the search for a natural antimicrobial agent. This study provides scientific understanding to further determine the antimicrobial values and investigate other pharmacological properties.

## 1. Introduction

Recently there has been a lot of attention focused on producing medicines and products that are natural. Several fruits and fruit extracts, as well as arrowroot tea extract [1] and caffeine [2], have been found to exhibit antimicrobial activity against *E. coli *O157:H7. This suggests that plants which manifest relatively high levels of antimicrobial action may be sources of compounds that can be used to inhibit the growth of foodborne pathogens. Bacterial cells could be killed by the rupture of cell walls and membranes and by the irregular disruption of the intracellular matrix when treated with plant extracts [1].

The guava (*Psidium guajava*) is a phytotherapic plant used in folk medicine that is believed to have active components that help to treat and manage various diseases. The many parts of the plant have been used in traditional medicine to manage conditions like malaria, gastroenteritis, vomiting, diarrhea, dysentery, wounds, ulcers, toothache, coughs, sore throat, inflamed gums, and a number of other conditions [3–5]. This plant has also been used for the controlling of life-changing conditions such as diabetes, hypertension, and obesity [3, 6–10]. In this study, we aim to evaluate the total extracts of *P. guajava *leaves, growing at Fort Valley State University, using various aqueous and organic solvents to establish if it is effective against killing or inhibiting the growth of foodborne bacterium *Staphylococcus aureus, Escherichia coli, Salmonella enteritidis*, and *Bacillus cereus* which can cause foodborne illness and spoilage.

**Figure 1:**
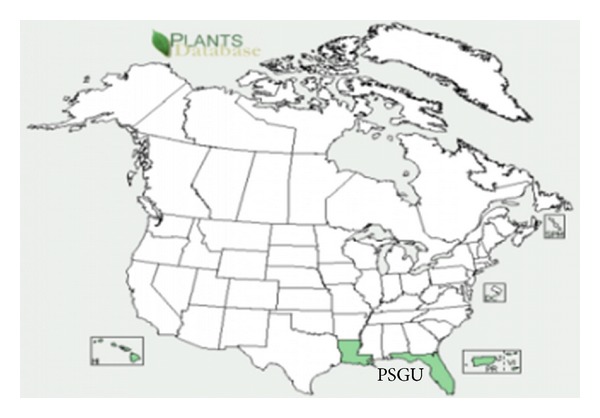
USDA plant database. distribution of guava in USA.

The genus *Psidium* belongs to the family Myrtaceae, which is considered to have originated in tropical South America. Guava crops are grown in tropical and subtropical areas of the world like Asia, Egypt, Hawaii, Florida (Figure 1), Palestine, and others. The genus *Psidium* comprises approximately 150 species of small trees and shrubs in which only 20 species produce edible fruits and the rest are wild with inferior quality of fruits [11]. The most commonly cultivated species of *Psidium* is *P. guajava *L. which is the common guava. Other species are utilized for regulation of vigor, fruit quality improvement and resistance to pest and disease [11]. Guava fruit today is considered minor in terms of commercial world trade, but it is widely grown in the tropics, enriching the diet of hundreds of millions of people in those areas of the world.

The guava tree is an evergreen small tree. The guava leaves are 2 to 6 inches long and 1 to 2 inches wide, aromatic when crushed, and appear dull-green with stiff but coriaceous with pronounced veins [12]. There are bioactive components in the guava leaf that can fight against pathogens, regulate blood glucose levels, and can even aid in weight loss. The leaves of guava contain an essential oil rich in cineol, tannins, triterpenes, flavonoids, resin, eugenol, malic acid, fat, cellulose, chlorophyll, mineral salts, and a number of other fixed substances [13–15].

The general techniques of medicinal plant extraction include maceration, infusion, percolation, digestion, decoction, Soxhlet extraction, aqueous-alcoholic extraction by fermentation, counter-current extraction, microwave-assisted extraction, ultrasound extraction, supercritical fluid extraction, and phytonic extraction. Maceration extraction is crude extraction; solvents diffuse into solid plant material and solubilize compounds with similar polarity [16]. Effect of plant material depends on its origin, variations in the extraction technique, the time, temperature of extraction, solvent concentration and polarity, quantity, and secondary metabolite composition of an extract [17]. Variations in extraction methods are usually found in the length of the extraction period, the solvent used pH, temperature, particle size, and the solvent-to-sample ratio [15].

Gonçalves et al. [18] conducted a study where they screened the antimicrobial effect of essential oils and methanol, hexane, and ethyl acetate extracts from guava leaves. The extracts were screened against bacteria strains isolated from seabob shrimp and laboratory culture strains. The guava leaves were extracted using a Soxhlet extractor and solvents in order of polarity and then concentrated in a rotary evaporator. The essential oil was obtained from fresh leaves of guava using a Clevenger type doser and the extraction methodology of Gottlieb and Magalhães [19]. The fresh leaves were submerged in distilled water in a 5 L glass bowl and submitted to the hydrodistillation technique for 24 h. The water and oil mixture were separated by drying with anhydrous sodium sulphate and then filtered. The extracts and the essential oil were evaluated by the disc diffusion method with the three extracts being tested at four concentrations. They found that the methanol extract showed greatest bacterial inhibition. No statistically significant differences were observed between the tested extract concentrations and their effect. The essential oil extract showed inhibitory activity against *S. aureus* and *Salmonella* spp. The researchers concluded that guava leaf extracts and essential oil are very active against *S. aureus*, thus making up important potential sources of new antimicrobial compounds.

 Antibacterial screening has been done selectively by many researchers in guava essential oil and solvent extract [1, 4, 20, 21]. The mechanism by which they can inhibit the microorganisms can involve different modes of action. It has been reported that these oils and extracts penetrate the lipid bilayer of the cell membrane, rendering it more permeable, leading to the leakage of vital cell contents [22, 23]. Sanches et al. [24] evaluated the antibacterial activities of *guava *against gram-positive and gram-negative bacteria testing ethanol and water extract of *P. guajava *leaves, stem, bark and root, and aqueous extract against *Staphylococcus aureus *were found to be more active by using ethanol and water extract than with just aqueous extract [1, 7]. Sacchetti et al. [25] reported that the oil showed a strong resistance against *Yarrowia lipolytica* which is a pathogenic yeast. Vieira et al. [26] have also reported the antibacterial effect of guava leaves extracts and found that they inhibited the growth of the *S. aureus*. Gnan and Demello [27] testing guava leaf extract found good antimicrobial activity against nine different strains of *Staphylococcus aureus*. The antibacterial activity of guava leaf extract was tested against acne developing organisms by Qa'dan et al. [28] concluding that the leaf extracts may be beneficial in treating acne especially when they are known to have anti-inflammatory activities.

Phytochemicals are nonnutritive chemicals produced by plants for their own protection, but they have been found to protect humans against diseases through recent research. Scientists have identified thousands of phytochemicals, although only small fractions have been studied closely and each one works differently [29]. Begum et al. [30] reported the isolation of two triterpenoids: guavanoic acid and guavacoumaric acid from the leaves of guava. Four flavonoids were isolated and identified by Arima and Danno [31] which were found to inhibit the growth of *Salmonella enteritidis* and *Bacillus cereus*. A study was done to evaluate the spasmolytic activity of guava leaf and was found that a compound called “aglycone quercetin” is responsible for spasmolytic activities, which is formed when flavonoids of guava leaves are hydrolyzed by the gastrointestinal fluids.

## 2. Materials and Methods

### 2.1. Preparation of Plant Extract

The leaf samples were collected from the guava trees growing at the Specialty Plant House at Fort Valley State University. Random leaf samples were collected into plastic zip lock bags with appropriate labeling and stored in an ice cooler until being transported to the laboratory for extraction.

### 2.2. Extraction Methods Used on Guava

The leaf samples were washed in tap water, dried, and placed into a blender to be grounded into powder. Four solvents were arranged in increasing polarity; n-hexane (>95%), methanol (>95%), ethanol (>99.5%), and boiling distilled water were used for the maceration extraction procedure. The leaf powder was added to each of solvents to make a 20% concentration. The mixtures were made in sterile 125 mL Erlenmeyer flask wrapped in aluminum foil to avoid evaporation and exposure to light for 3 days at room temperature. The flasks were placed on a platform shaker at 70 rpm. After 3 days of soaking in solvent, the mixtures were transferred to 50 mL tubes and centrifuged for 10 min at 4,000 rpm at 25°C. The supernatant was collected and stored at 4°C until use.

### 2.3. Phytochemical Analysis

Chemical tests for the screening and identification of bioactive chemical constituents in the guava were carried out with the extracts using the standard procedure as described [32–34]. For each test, 1 mL of each solvent extract was used for analysis, in exception for the saponin test in which 3 mL solvent extract was used.

### 2.4. Test for Saponins

**Figure 2:**
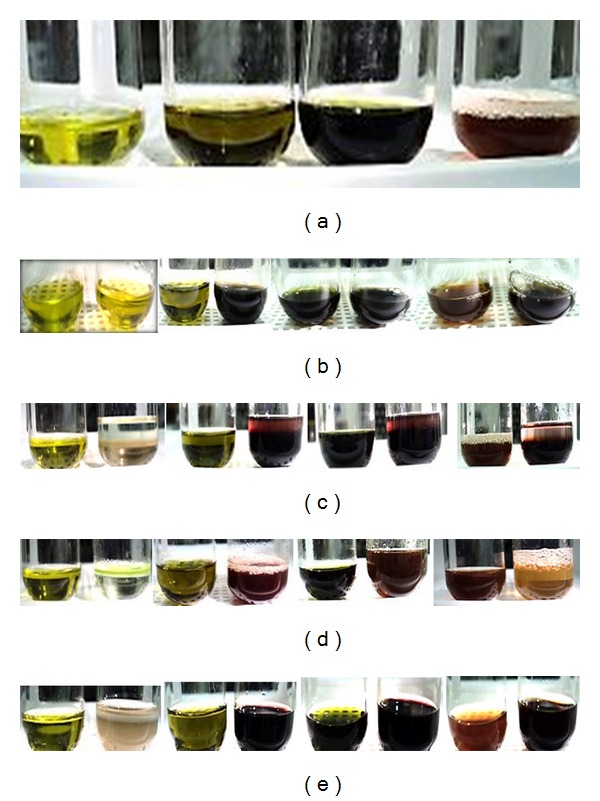
(a) Existence of saponin tests; L to R: n-hexane, methanol, ethanol, and distilled water extracts. (b) Existence of phenols and tannins tests; L to R: n-hexane, methanol, ethanol, and distilled water extracts. (c) Existence of terpenoids tests; L to R: n-hexane, methanol, ethanol, and distilled water extracts. (d) Existence of flavonoids tests; L to R: n-hexane, methanol, ethanol, and distilled water extracts. (e) Existence of glycosides tests; L to R: n-hexane, methanol, ethanol, and distilled water extracts.

Extract was placed in a test tube and shaken vigorously. The formation of stable foam was taken as an indication for the presence of saponins (Figure 2(a)).

### 2.5. Test for Phenols and Tannins

Extract was mixed with 2 mL of 2% solution of FeCl_3_. A blue-green or black coloration indicated the presence of phenols and tannins (Figure 2(b)).

### 2.6. Test for Terpenoids (Salkowski's Test)

Extract was mixed with 2 mL of chloroform. Then 2 mL of concentrated sulfuric acid was added carefully and shaken gently. A reddish brown coloration of the interphase was formed to show positive results for the presence of terpenoids (Figure 2(c)).

### 2.7. Test for Flavonoids (Shinoda Test)

Extract was mixed with magnesium ribbon fragments, and concentrated hydrochloric acid was added drop wise. Orange, red, pink, or purple coloration indicates the presence of flavonoids (Figure 2(d)).

### 2.8. Test for Glycoside

Extract was mixed with 2 mL of glacial acetic acid containing 2 drops of 2% FeCl_3 _. The mixture was poured into another tube containing 2 mL of concentrated sulfuric acid. A brown ring at the interphase indicates the presence of glycosides (Figure 2(e)).

### 2.9. Panel of Microorganisms

A board of organisms comprising 2 Gram-negative bacteria, *Escherichia coli* (*Escherichia coli* B, Living Bacteriophage Host, item no. 124300) and *Salmonella enteritidis* (*Salmonella enteritidis*, MicroKwik Culture, Pathogen, item no. 155350A), and 2 Gram-positive bacteria, *Staphylococcus aureus* ((*Staphylococcus aureus,* coagulase positive), MicroKwik Culture, Pathogen, item no. 155554A) and *Bacillus cereus *(*Bacillus cereus,* Living, item no. 154872) was selected to test the guava extracts ability to inhibit the growth. All strains were purchased from Carolina Biological Supply Company, (Burlington, NC 27215-3398, USA). Prior to sensitivity testing, each of the bacteria strains were cultured onto nutrient agar plates and incubated for 18 to 24 h at 37°C to obtain colonies. After overnight incubation, colonies were selected with a sterile disposable inoculating loop and transferred to a glass tube of sterile physiological saline and vortex thoroughly. Each bacterial suspension turbidity is then compared to that of the 0.5 McFarland standard solution (containing about 1.5 × 10^8^ CFU/mL).

### 2.10. Antibacterial Activity

Antimicrobial susceptibility testing was done using the well-diffusion method according to the standard of the National Committee for Clinical Laboratory Standards [35]. The plant extracts were tested on Mueller Hinton II plates to detect the presence of antibacterial activity. Prior to streaking the plates with bacteria, 5 mm diameter wells were punched into the medium using a sterile borer. All plates were inoculated with the test bacterium which has been previously adjusted to the 0.5 McFarland standard solution; a sterile cotton swab was dipped into the suspension, rotated several times, and pressed firmly on the inside wall of the tube above the fluid level removing excess inoculum. The surface of the agar plate was streaked over the entire sterile agar surface rotating the plate to ensure an even distribution of inoculum with a final swab around the rim. The plates are allowed 3 to 5 min to dry the excess moisture. Fifty uL aliquots of each test extract was dispensed into each well after the inoculation of the plates with bacteria. The wells were also arranged in a triangle formation 2 inches apart. The same extract was used on each plate, with a total of three plates used for each extract for selecting bacterium. For each bacterial strain, controls were maintained where pure solvents were used instead of the extract. The plates are sealed with parafilm, labeled, and placed in an incubator set to 37°C. After 24 hours of incubation, each plate was examined for inhibition zones. A ruler was used to measure the inhibition zones in millimeters. Every experiment was carried out in parallel, and the results represented the average of at least three independent experiments.

## 3. Results and Discussions

### 3.1. Phytochemical Analysis

Table 1 shows the summarized phytochemical screening of chemical constituents of guava extracts under study on qualitative basis. The results revealed the presence of active compounds in the four different extracts. As the table shows, the methanol and ethanol extracts indicate the presence of tannins, phenols, flavonoids, terpenoids, and glycosides, but absence of saponins. Distilled water is the only that showed the presence of all the phytochemicals, whereas solvent n-hexane failed to have any of the chemical compounds present.

The analysis of the plant extracts revealed the presence of phytochemicals which are known to exhibit medical and physiological activities. For example, tannins are polyphenolic compounds that bind to proline rich protein that interferes with protein synthesis [24, 36, 37] and has shown to have antibacterial activity [38, 39]. Flavonoids are hydroxylated polyphenolic compounds known to be produced by plants in response to microbial infections to which this aspect has been extensively studied and found to have antimicrobial activity against an array of microorganisms *in vitro *[40]. Their ability has been attributed to their ability to form complexes with extracellular and soluble proteins and bacterial cell walls [41]. Terpenoids although mainly used for their aromatic qualities have also been found to be potential agents against inhibiting bacteria [36]. Saponins which are glycosides have been found to have inhibitory effects on gram-positive organism, *S. aureus*. Therefore, the phytochemical analysis revealed that the methanol, ethanol, and distilled water extract have chemical compounds that have been found to possess antibacterial activities, which could contribute to the results obtained from antibacterial analysis. Figures 2(a) to 2(e) show the colorimetric results for the solvent extracts.

### 3.2. Antibacterial Activity

The results of the study indicated that only two of the crude solvent extracts prepared from the leaves of *Psidium guajava*, methanol and ethanol, showed inhibitory activity against bacteria (Table 2). Only Gram-positive bacteria, *Bacillus cereus* and *Staphylococcus aureus,* were susceptible to the two extracts, while neither of the Gram-negative bacterium showed any inhibition. At 10 mg/50 *µ*L, the methanol extract had a slightly higher antibacterial activity with mean zones of inhibition 8.27 and 12.3 mm than ethanol extract with mean zone of inhibition 6.11 and 11.0 mm against *B. cereus* and *S. aureus*, respectively. The resistance of the Gram-negative bacteria could be attributed to its cell wall structure. Gram-negative bacteria have an effective permeability barrier, comprised of a thin lipopolysaccharide exterior membrane, which could restrict the penetration of the extruding the plant extract. It has been reported earlier that Gram-negative bacteria are usually more resistant to the plant-origin antimicrobials and even show no effect, compared to Gram-positive bacteria [42–44]. Gram positive bacteria have a mesh-like peptidoglycan layer which is more accessible to permeation by the extracts [22, 28, 42, 43].

Results found in this study were supported and/or opposed in the data reported in literature. Nascimento et al. [45] conducted a study which supports the finding of the present study in which the guava extract was able to have inhibitory effects against *Staphylococcus *and *Bacillus *and no effect on the *Escherichia *and *Salmonella, *whereas Chanda and Kaneria [46] oppose the findings concerning the Gram-negative bacteria. Mahfuzul Hoque et al. [21] found no antibacterial activity of ethanolic extracts of guava against *E. coli* and *S. entertidis*; however Vieira et al. [26] found guava sprout extracts were effective against inhibiting *E. coli*.

Sanches et al. [24] found that the aqueous extract of guava was effective against *Staphylococcus* and *Bacillus*. The methanolic extracts of guava reported by Lin et al. [47] showed significant inhibitory activity against the growth of 2 isolates of *Salmonella* and enteropathogenic *E. coli*.

## 4. Conclusions

The present work demonstrates the antimicrobial potential of *Psidium guajava* leaves extract by using various solvents. The results indicate that ethanol and methanol are better than n-hexane and water for the extraction of the antibacterial properties of guava. The results also indicate that the plant extracts have no antibacterial effect on the Gram-negative bacteria, showing that they do not contain active ingredients against the organisms. The observed inhibition of Gram-positive bacteria, *Bacillus cereus *and *Staphylococcus aureus, *suggests that guava possesses compounds containing antibacterial properties that can effectively suppress the growth when extracted using methanol or ethanol as the solvent. Comparisons with related data from the literature indicate that according to the different methodologies of studies on antibacterial activity, the most diverse outcomes can be obtained. This study provides scientific insight to further determine the antimicrobial principles and investigate other pharmacological properties of guava. On the basis of the present finding, *P. guajava* leaves possess the capabilities of being a good candidate in the search for a natural antimicrobial agent against infections and/or diseases caused by *B. cereus* and *S. aureus*.

## Figures and Tables

**Table 1:** Phytochemical constituents of *Psidium guajava* extracts.

Extracts	Phenols and tannins	Saponins	Terpenoids	Flavonoids	Glycosides
n-Hexane	−	−	−	−	−
Methanol	+	−	+	+	+
Ethanol	+	−	+	+	+
Distilled water	+	+	+	+	+

+: presence of constituent (positive); −: absence of constituent (negative).

**Table 2:** Antibacterial activity of *Psidium guajava* leaves of the screened solvents extracts.

Plant extracts	Zone of inhibition*(mm)			
	*B. cereus *	*S. aureus *	*E. coli *	*S. entertidis *
n-Hexane	—	—	—	—
Ethanol	6.11 ± 0.60	11.0 ± 0.52	—	—
Methanol	8.27 ± 0.44	12.3 ± 0.78	—	—
Water	—	—	—	—

*Inhibition zones are the mean including borer (5 mm) diameter ± standard deviation.

—: no inhibitory activity.
